# Supplementary material for: Non-Steroidal Anti-Inflammatory Drugs (NSAIDs): Usage and co-prescription with other potentially interacting drugs in elderly: A cross-sectional study
Source: PLoS One. 2020 Oct 9;15(10):e0238868. doi: 10.1371/journal.pone.0238868 (PMC7546451; doi:10.1371/journal.pone.0238868)
Supplement: S1 File — (PDF) [file pone.0238868.s001.pdf]

Data recording form

Non-steroidal Anti-Inflammatory Drugs (NSAIDs): Usage and Co-prescription with Other Potentially Interacting Drugs in Elderly

Area Identification			
Hospital		☐☐	
Patient Number		☐☐☐	
**Section A: Socio-demographic information and background characteristics**			
**No.**	**Questions and Filter**	**Coding Categories**	**Skip**
101	Age (in completed years)	☐☐☐	
102	Sex	Male…………………………1<br>Female………………………2	
103	Marital status	Married……………….…….1<br>Single……………………......2<br>Divorced……………….........3<br>Widowed……….………….…4	
104	Educational level	No formal education………..1<br>Primary (1-5)……………….…2<br>Middle (6-8)………………...3<br>Secondary (9-12)…………....4<br>Higher…………………….........5	
105	Occupation	Governmental……………….1<br>Private service………….......2<br>Self-employed………….......3<br>Unemployed………………...4<br>House wife…………………..5	
106	Chronic illness	Hypertension……………….A<br>Diabetes…………………….B<br>Asthma……………………...C	

	(Multiple answers are possible)	Renal failure………………..D<br>Rheumatoid arthritis………..E<br>Others (specify)__________X	
107	History of Gastro-intestinal (GI) upset	Yes…………………………..1<br>No…………………………...2<br>I don't remember……….......3	
108	Religion	Christian…………………….1<br>Muslim………………….......2<br>Others (specify) __________3	
109	Ethnic group	Tigrigna……………………..1<br>Tigre………………………...2<br>Bilen………………………...3<br>Saho…………………………4<br>Afar……………………........5<br>Kunama……………………..6<br>Nara……………………........7<br>Hidarb……………………….8<br>Rashaida…………………….9	
**Section B: Usage of Gastro-protective agents (GPAs) among chronic NSAID users**			
201	Have you ever taken NSAID before?	Yes…………………………..1<br>No…………………………...2<br>I don't know………………...3	} Section C
202	For how long have you been taking NSAID?	Duration in weeks<br>_______________	
203	Have you been taking gastro-protective agent(s) along with the NSAID?	Yes………………………….1<br>No…………………………..2<br>I don't know………………..3	} 205
204	What is the GPA(s) that you have been taking?	Omeprazole…………………1<br>Famotidine………………….2<br>Ranitidine…………………...3<br>Others (specify)___________4	

205	Have you experienced any Adverse Drug Reaction(s) (ADRs) as a result of chronic NSAID use?	Yes…………………………..1<br>No…………………………...2	→ Section C
206	Which of the following ADRs do you encounter?<br>***Multiple answers are possible***	Gastro-intestinal (GI) upset...A<br>GI ulcer...…...………………B<br>Headache……………...……C<br>Others (specify) ___________X	
**Section C: Prescription pattern analysis of NSAIDs<br>(Extract the following information from prescription)**			
301	Type of NSAID(s) prescribed<br>***(Multiple answers are possible)***	Aspirin tablet……………….A<br>Diclofenac sodium tablet…...B<br>Diclofenac sodium gel……...C<br>Diclofenac sodium injection……………………D<br>Diclofenac sodium suppository………………….....E<br>Ibuprofen tablet……………..F<br>Indomethacin tablet………...G<br>Indomethacin suppository….H<br>Others (specify)___________X	
		**Drug 1**	**Drug 2**
302	Dose and Frequency		
303	Duration (in days)		
304	Route of administration		
305	Dosage form		
306	Total number of drugs per prescription	☐☐	
307	Total number of NSAID(s) per prescription	☐☐	
**Section D: Analysis of NSAID drug interactions with other concurrently used drugs**			

No.	Questions and Filter		Coding Categories	Skip
401	Drug(s) prescribed along with NSAID(s)		1. ____________<br>2. ____________<br>3. ____________	
402	Are you taking presently any drug(s) as self-medication?		Yes……………………1<br>No……………………..2	→ 405
403	Specify self-medicated drug		1. ___________<br>2. ___________<br>3. ___________	
404	**Analysis of NSAID drug interactions with self-medicated drugs**			
	**Drug interactions**		**Severity**	**Clinical implication**
	**Drug 1 (NSAID)**	**Drug 2**		
404A			Severe………..………1<br>Moderate……….………2<br>Mild……………..………3<br>No interaction…..………4	
404B			Severe………..………1<br>Moderate……….………2<br>Mild……………..………3<br>No interaction…..………4	
404C			Severe………..………1<br>Moderate……….………2<br>Mild……………..………3<br>No interaction…..………4	
404D			Severe………..………1<br>Moderate……….………2<br>Mild……………..………3<br>No interaction…..………4	
405	**Analysis of NSAID drug interactions with other prescribed drugs**			

	Drug interactions		Severity	Clinical implication
	Drug 1 (NSAID)	Drug 2		
405A			Severe………..………1<br>Moderate……….………2<br>Mild…………….………3<br>No interaction…..………4	
405B			Severe………..………1<br>Moderate……….………2<br>Mild…………….………3<br>No interaction…..………4	
405C			Severe………..………1<br>Moderate……….………2<br>Mild…………….………3<br>No interaction…..………4	
405D			Severe………..………1<br>Moderate……….………2<br>Mild…………….………3<br>No interaction…..………4	
**Section E: Medical card review<br>(Extract the following information from medical cards)**				
501	Indication (s) for NSAID prescription<br><br>***(Multiple answers possible)***		Backache………………A<br>Arthritis………………..B<br>Anti-platelet effect…….C<br>Knee pain……………...D<br>Leg pain……………….E<br>Others (specify)_______F	
502	Co-morbidity<br>***(Multiple answers possible)***		Hypertension…………..A<br>Diabetes…..…………....B<br>Asthma.………………..C<br>Renal failure…..…...…..D	

		Others (specify)_______X	
503	History of peptic ulcer	Yes………..……………1<br>No…………..…………2	

Name of data collector **Date of data collection**

__________________________________ ____________________

Comment section
